# Mutational Analysis of the Respiratory Nitrate Transporter NarK2 of *Mycobacterium tuberculosis*

**DOI:** 10.1371/journal.pone.0045459

**Published:** 2012-09-18

**Authors:** Michelle M. Giffin, Ronald W. Raab, Melissa Morganstern, Charles D. Sohaskey

**Affiliations:** 1 Department of Veterans Affairs Medical Center, Long Beach, California, United States of America; 2 ISAT Department, James Madison University, Harrisonburg, Virginia, United States of America; Tulane University, United States of America

## Abstract

*Mycobacterium tuberculosis* induces nitrate reductase activity in response to decreasing oxygen levels. This is due to regulation of both the transcription and the activity of the nitrate transporter NarK2. A model of NarK2 structure is proposed containing 12 membrane spanning regions consistent with other members of the major facilitator superfamily. The role of the proton gradient was determined by exposing *M. tuberculosis* to uncouplers. Nitrite production decreased indicating that the importation of nitrate involved an H^+^/nitrate symporter. The addition of nitrite before nitrate had no effect, suggesting no role for a nitrate/nitrite antiporter. In addition the NarK2 knockout mutant showed no defect in nitrite export. NarK2 is proposed to be a Type I H^+^/nitrate symporter. Site directed mutagenesis was performed changing 23 amino acids of NarK2. This allowed the identification of important regions and amino acids of this transporter. Five of these mutants were inactive for nitrate transport, seven produced reduced activity and eleven mutants retained wild type activity. NarK2 is inactivated in the presence of oxygen by an unknown mechanism. However none of the mutants, including those with mutated cysteines, were altered in their response to oxygen levels. The assimilatory nitrate transporter NasA of *Bacillus subtilis* was expressed in the *M. tuberculosis* NarK2 mutant. It remained active during aerobic incubation showing that the point of oxygen control is NarK2.

## Introduction

Nitrate can serve as a terminal electron acceptor as well as a source of nitrogen for many bacteria. The first step for either of these processes involves the active transportation of nitrate across a membrane into the cell where it is reduced to nitrite. Nitrate, a charged molecule, must rely on a transporter to be efficiently imported. Nitrate transport has been studied in a variety of microbes. Transport during nitrate respiration has mostly focused on gram negative bacteria such as *E. coli* and *Paracoccus* sp. For the purpose of assimilation *Aspergillus nidulans* has been the model organism. Nitrate is a major source of nitrogen for higher plants including many crops. Thus the topic is of economic and medical importance.

The major facilitator superfamily consists of transporters found in all kingdoms of life [1]. Family 8 (TC 2.A.1.8) of this superfamily comprises the nitrate/nitrite porter proteins (NNP). Phylogenetic analysis of the bacterial NNPs further identified two subgroups [2], [3]. Type I were proposed to be H^+^/NO_3_^−^ symporters that relied on the proton gradient for activity. Type II were nitrate/nitrite antiporters that had low activity in the absence of nitrite [4].

*Mycobacterium tuberculosis,* the causative agent of tuberculosis, is an obligate aerobe. During the course of infection it faces many stressful conditions including hypoxia [5], [6]. When oxygen becomes limiting *M. tuberculosis* ceases replication and enters a nonreplicating persistent (NRP) state which is proposed to be similar to that seen in latent tuberculosis [7], [8]. Nitrate reductase activity is induced which provides energy during this transition [9], [10]. By maintaining the proton gradient, nitrate reductase enhances the survival of *M. tuberculosis* during exposure to nitric oxide or the combined effects of hypoxia and carbon starvation, or hypoxia and acid [11], [12]. A nitrate reductase mutant of the close relative *Mycobacterium bovis* BCG showed reduced virulence and reduced lung damage in SCID mice. In immunocompetent mice the defect was less pronounced although there was decreased persistence in the lungs, liver and kidneys [13], [14].

The nitrate reductase enzyme is encoded by *narGHJI* and is constitutively expressed in *M. tuberculosis*. Enzyme levels are independent of the presence of oxygen, nitrate and nitrite [15]. The increase in nitrate reductase activity during hypoxic NRP is not due to induction of the nitrate reductase enzyme, but rather to increased levels of the nitrate transporter encoded by *narK2* [15]. Transcription of *narK2* is induced by hypoxia but independent of nitrate or nitrite. This pattern of expression is true *in vivo* also, as *narK2,* but not *narGHJI,* was induced during chronic infection in mice [16]. There is additional regulation as the activity of NarK2 is inhibited by oxygen [17]; a general feature of hypoxic nitrate transporters [15], [18]–[20]. This effect is not due to the presence of molecular oxygen but rather to the redox state of the cell [17], [18], [21].

NarK2 plays an important role in the dormancy in *M. tuberculosis* given that the main point of control for nitrate respiration is the transport of nitrate. Therefore we set out to further characterize the function of NarK2. To gain insight into the mechanism of nitrate transport NarK2 was analyzed for both H^+^/NO_3_^−^ symporter, and nitrate/nitrite antiporter activity. Site-directed mutagenesis of conserved amino acids identified important regions and residues of the protein.

## Materials and Methods

### Culture Conditions

*M. tuberculosis* H37Rv was grown in Dubos Tween-albumin broth (DTA, Difco, Detroit, MI). Growth was monitored by measuring the OD_580_ in a Coleman model 35 spectrophotometer (Coleman Instruments, Maywood, IL). Cultures were started with an initial density of 2.5×10^6^ cells/ml. Aerobic cultures were incubated at 37°C on a model G24 rotary shaker-incubator at a speed of 225 rpm (New Brunswick Scientific Co. Inc, Edison, NJ).

For microaerobic and anaerobic cultures the Wayne model was used with culture tubes sealed with Wheaton red rubber septum caps (Fisher Scientific, Pittsburgh, PA) and wrapped with parafilm [8]. After approximately 67 hrs growth stopped and cultures of *M. tuberculosis* entered the microaerobic nonreplicating persistent state I (NRP-1). Fully anaerobic NRP-2 was reached after roughly 200 hrs of incubation.

### Treatment with Protonophores

NRP-1 cultures, containing approximately 10^8^ cells/ml were opened and pooled. Aerobic cultures were diluted in DTA to the same cell concentration based on optical density (OD_580_ 0.1 = 6.25×10^7^ CFU/ml). NaNO_3_ was added to 5 mM. The uncouplers (Sigma, St. Louis, MO) in water were added. 2,4-dinitrophenol (DNP) was used at 1 mM, carbonyl cyanide *p*-trifluoromethoxyphenylhydrazone (FCCP) was at 200 µM. Cultures were dispensed into tubes in triplicate. For aerobic conditions tubes were loosely capped and incubated with shaking. For anaerobic conditions cultures were filled to the top and Oxyrase was added to remove oxygen (Oxyrase Inc Mansfield, OH). For some tubes NaNO_2_ and Oxyrase were added and then incubated at 37°C for 4 hrs before the nitrate was added. Nitrite concentrations were measured at 2.5 hrs intervals. To measure oxygen utilization methylene blue was added to 0.0003% and the cultures were transferred to 8 ml culture tubes and tightly capped [17]. Decolorization of methylene blue was monitored by measuring absorbance at 665 nm.

### Detection of Nitrite in Cells

Cultures of RVW3 (Δ*narK2*) were grown to NRP-1 (115 h). The cells were centrifuged and resuspended in the same volume of DTA with 5 mM NaNO_3_ (also containing 5 mM benzyl viologen where indicated) at either pH 6.7 or 8.5. 500 µl of culture was transferred to 0.65 ml graduated microtubes (in triplicate). The reaction was initiated with the addition of 120 µl of 60 mM dithionite in 10 mM NaOH which reduced the benzyl viologen. To a control set of tubes 120 µl of 10 mM NaOH was added (oxidized benzyl viologen samples). After 1.5 hrs incubation at 37°C the tubes were centrifuged. The medium was removed while the cells were treated for 10 min in 1 M HCl. Nitrite levels were then determined in both the medium and cells fractions.

RVW3 pNarK2 and RVW3 pNasA were grown to NRP-1 in the Wayne model. They were treated as above except instead of benzyl viologen, Oxyrase was added. After 3 hrs cells were pelleted by centrifugation and the medium carefully removed.

### Cloning of *nasA* in *M. Tuberculosis*

The *nasA* of *Bacillus subtilis* was amplified with the primers pNasA-F and pNasA-R (Table S1). Two reactions were run and the 1310 bp fragment from each was cloned and sequenced. These were compared to the published *nasA* sequence [22] . Both of the new sequences differed from the published *nasA* sequence by the same base. This change would result in a NasA protein that was 20 amino acids longer than previous reported. The longer NasA was more similar to NasA sequences from other *Bacillus* species and was verified as correct by additional PCR and sequencing.

The promoterless *nasA* was inserted into the integrating plasmid pMP102 in front of the *narK2* promoter by digesting both plasmids with *Eco*RI and *Kpn*I [15]. A hygromycin resistance marker was inserted into the *Sma*I site to make pNasA. This plasmid was electroporated into *M. tuberculosis* RVW3 (Δ*narK2*) with selection for resistance to hygromycin. The *nasA* insertion was verified by Southern analysis.

### Mutagenesis of *narK2*

All primers were from Sigma-Aldrich (Table S1). The *narK2* complementing plasmid pNarK2 was used for mutagenesis [23]. This plasmid was used for site directed mutagenesis using the QuikChange® II XL Site-Directed Mutagenesis Kit (Stratagene, Santa Clara, CA). After mutagenesis the plasmids were transformed into *E. coli* with selection for gentamycin resistance. Plasmids containing the mutation were identified by sequencing. Each plasmid was then electroporated into *M. tuberculosis* RVW3 (Δ*narK2*) followed by selection for gentamycin resistance [15]. Southern analysis was performed to verify the presence of both the inactive wild type gene and the presence of the mutated gene. PCR was use to amplify the mutated *narK2* using primers p173 and p174 (Table S1) and the product sequenced to verify the presence of the correct mutation.

To measure nitrite production cultures were grown in DTA with 5 mM NaNO_3_ for 115 hrs in shaking cultures (aerobic), 115 hrs in the Wayne model (microaerobic NRP-1) or 255 hrs in the Wayne model (anaerobic NRP-2). Aerobic cultures were in mid-logarithmic phase (OD_580_∼0.4) whereas NRP-1 cultures were hypoxic for approximately 48 hrs [8]. Samples were removed and nitrite concentrations determined by the Griess reaction.

## Results

### Role of the Proton Gradient

During growth in DTA medium *M. tuberculosis* reduces nitrate to nitrite but does not reduce nitrite further. The nitrite accumulates in the extracellular medium and can be used as a sensitive method to monitor the combined activity of nitrate transport and its reduction. Aerobic cultures of *M. tuberculosis* do not expresses *narK2* and have only a low level of nitrate reductase activity resulting from diffusion of nitrate into the cell [15], [17]. In the Wayne model for dormancy *narK2* is induced at the beginning of microaerobic NRP-1 and maintained at a high level in anaerobic NRP-2 [15], [16].

**Figure 1:**
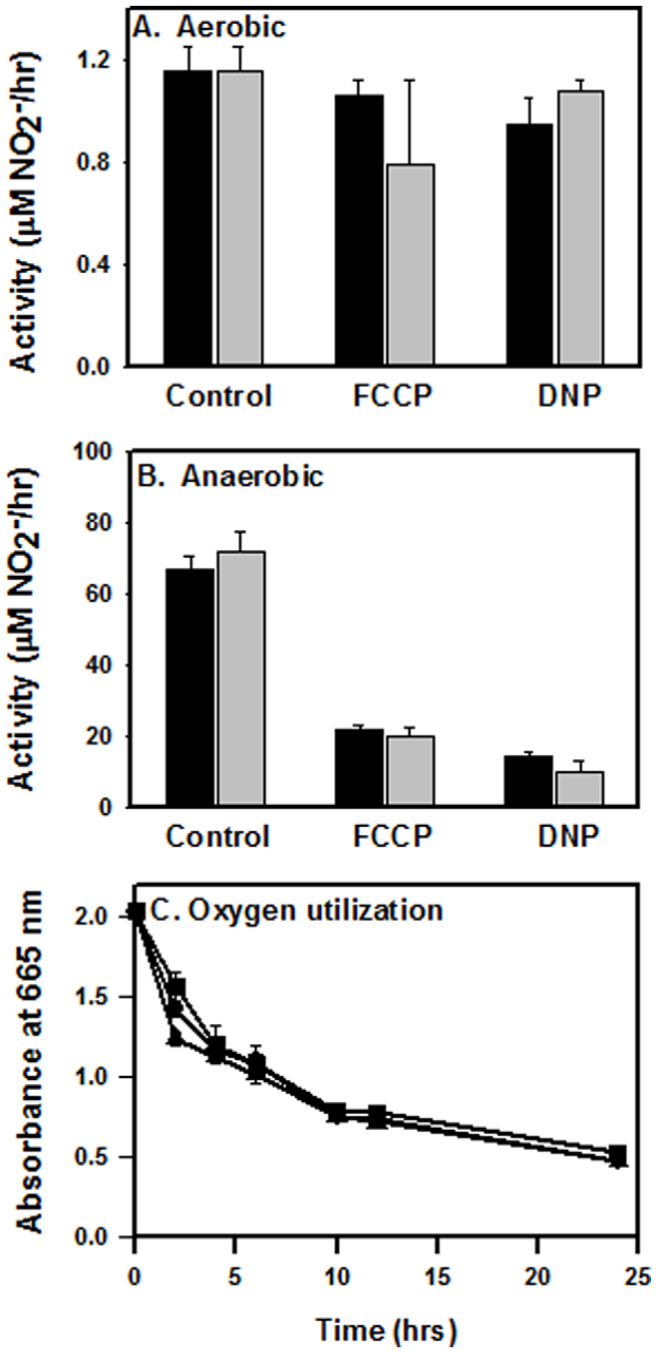
Effect of uncouplers on nitrite production. Cultures were grown either (A) aerobically or (B) to NRP-1 in the Wayne model. Each culture was then adjusted to a similar cell density. Nitrate was added along with uncouplers where indicated. Nitrite concentrations were measured at 2.5 hrs intervals. Black bars are without nitrite, while grey bars are from experiments following 2 hrs of preincubation with 50 µM NO_2_^−^. (C) *M. tuberculosis* was grown to mid-exponential phase and then divided for further incubation in full sealed tube with 0.0003% methylene blue (circles) and either DNP (squares) or FCCP (triangles). Standard deviation is indicated.

Uncouplers were used to determine if the proton gradient plays a role in NarK2-mediated import of nitrate. The effect of two different uncouplers on the nitrate reductase enzyme was first measured. FCCP and DNP were added to aerobic cultures of *M. tuberculosis* and nitrite levels measured. FCCP and DNP had little effect on nitrite production indicating no role for the proton gradient in the reduction of nitrate under aerobic conditions (Figure 1A).

The effect of each of these compounds on nitrite production by hypoxic *narK2*-expressing cultures was next determined. *M. tuberculosis* was grown to NRP-1 and both nitrate and an uncoupler were added. FCCP and DNP both decreased the rate of nitrite production indicating a role for the protein gradient in nitrate transport by NarK2 (Figure 1B). In aerobic and anaerobic cultures preincubation with 50 µM NO_2_^−^ did not alter the effect of the uncouplers. A decrease in the proton gradient could also reduce glucose uptake which could limit energy production. To verify that the decreased nitrite production is due to the inhibition of nitrate transport and not of metabolism, oxygen utilization was measured. The rate of methylene blue decolorization was used to monitor oxygen levels in the presence of each uncoupler (Figure 1C). No inhibition of respiration was seen.

### NarK2 does not Function as a Nitrate/nitrite Antiporter

**Figure 2:**
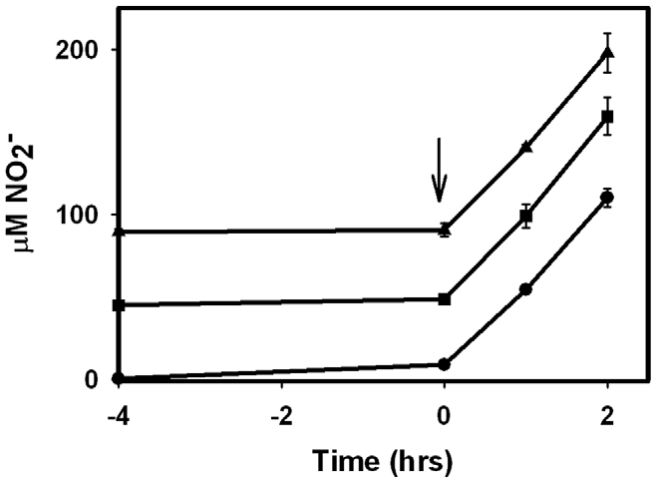
Effect of pretreatment with nitrite on nitrate reductase activity. *M. tuberculosis* was incubated anaerobically with 0, 50 or 100 µM NO_2_^−^. After 4 hrs nitrate was injected and nitrite levels measured at time 0, 1 and 2 h. The arrow indicates the time of the addition of nitrate. Circles – control, no nitrite. Squares – with 50 µM NO_2_^−^. Triangles – with 100 µM NO_2_^−^. Standard deviation is shown.

To determine if NarK2 functions as a nitrate/nitrite antiporter, the effect of nitrite on the initial rate of nitrate reduction was determined. Upon the addition of nitrate NNP antiporters have low activity until sufficient nitrate diffuses into the cell to provide nitrite for activity. This lag can be eliminated by providing nitrite in advance [4]. *M. tuberculosis* was grown in the Wayne model and cultures were aliquoted to tubes with either no addition, or nitrite at either 50 µM or 100 µM. These samples were incubated anaerobically with Oxyrase at 37°C. After 4 hours of pretreatment to allow nitrite to enter the cells, nitrate was injected and nitrite concentrations were determined 1 and 2 hours after the addition (Figure 2). Without pretreatment cells produced nitrite at a rate of 51 µM/hr. With the 50 µM NaNO_2_^−^ pretreatment the rate was similar, 55 µM/hr, and with 100 µM NaNO_2_ pre-treatment the rate was 54 µM/hr. No differences were seen in the rate of nitrite production between each sample suggesting NarK2 is not a nitrate/nitrite antiporter.

**Figure 3:**
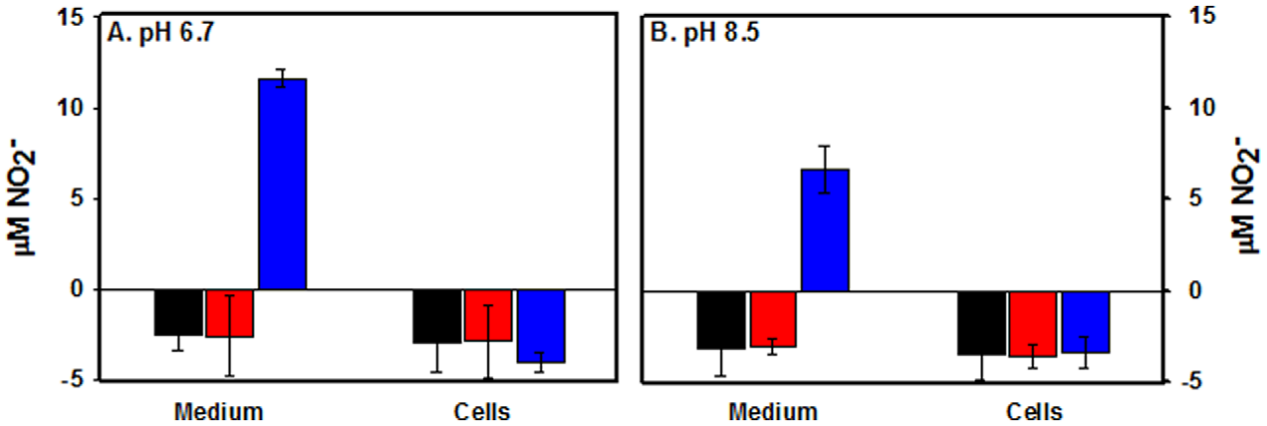
Export of nitrite in the absence of NarK2. Aerobic cultures of RVW3 were incubated with nitrate at pH 6.7 (A) or pH 8.5 (B). Untreated controls – black bars. With oxidized benzyl viologen – red bars, or with dithionite-reduced benzyl viologen – blue bars. Nitrite levels were measured after 90 min in medium or in cells. The standard deviation is indicated.

To detect a possible interaction between NarK2 and nitrite, the export of nitrite was analyzed. Benzyl viologen in the reduced state is a nitrate and nitrite ionophore and can transport nitrate into the cell [24]. Nitrite production by the *M. tuberculosis narK2* knockout mutant RVW3 was determined at pH 8.5 which would reduce the diffusion of both ions, and at the standard pH of 6.7. After incubation with nitrate and benzyl viologen the cells were separated from the medium and nitrite levels determined in both fractions (Figure 3). Without benzyl viologen or with the oxidized form there was no production of nitrite due to the defect in nitrate transport. When reduced benzyl viologen was added the nitrite levels in the medium increased. All nitrite was detected in the media, and none was associated with the cells indicating that the loss of *narK2* did not affect the ability of *M. tuberculosis* to excrete nitrite.

For an independent approach NasA of *Bacillus subtilis* was expressed in *M. tuberculosis*. NasA is an assimilatory Type I nitrate transporter closely related to NarK2 of *M. tuberculosis* [22]. During assimilation nitrate is transported into *B. subtilis* where it is reduced to nitrite before being assimilated in the cytoplasm. Nitrite is not excreted before assimilation. *nasA* from *B. subtilis* under control of the *M. tuberculosis narK2* promoter was integrated into the chromosome of *M. tuberculosis* in RVW3. As a control RVW3 expressing *narK2* from the same plasmid was used. RVW3 complemented with *narK2* showed low activity in aerobic cultures with strong induction in NRP-1 and NRP-2, in a manner very similar to wild type (Table 1). RVW3 expressing *nasA* produced similar levels of nitrite in NRP cultures indicating that NasA was able to complement the *narK2* knockout.

**Table 1:** Nitrite levels in *M. tuberculosis* expressing *B. subtilis nasA* or *M. tuberculosis narK2.*

	AG^a^	NRP-1^b^	NRP-2^c^
RVW3 pNarK2	133±8	1013±36	2689±28
RVW3 pNasA	258±6	925±14	2414±368

Mean nitrite concentration (micromolar) ± standard deviation.

a After 115 h of growth (final OD_580_ of ∼0.4).

b After 115 h in the Wayne model.

c After 255 h in the Wayne model.

RVW3 expressing either *narK2* or the *B. subtilis nasA* from the integrated plasmid were grown in the Wayne model to induce the expression of both genes. The cells were centrifuged, resuspended in media at pH 8.5 and nitrate added. The cultures were incubated anaerobically for 3 hrs, the cells separated from the medium and nitrite levels determined. Similar levels of nitrite were detected in the cells of both strains; 0.7 µM ±0.3 for RVW3 pNarK2, and 0.8 µM ±0.3 for RVW3 pNasA indicating that export was not inhibited in the absence of *narK2*.

### Mutagenesis of NarK2

**Figure 4:**
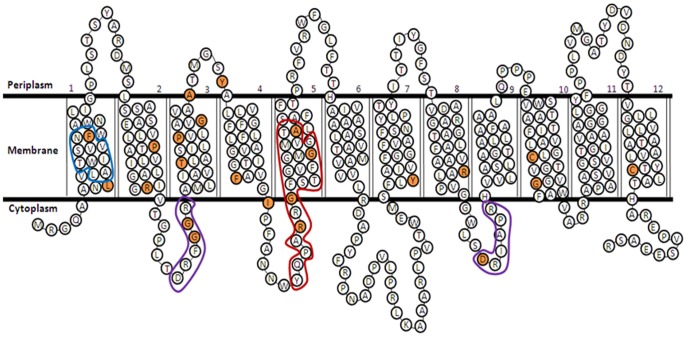
Model of NarK2 secondary structure. The predicted membrane spanning regions are shown. Amino acids that were changed are highlighted in orange. The conserved MFS motif is outlined in purple, the nitrate signature in red, and the eukaryote region in blue.

NarK2 of *M. tuberculosis* is a member of the major facilitator superfamily. Six computer models, DAS, HMMTOP, PREDTMR, SPLIT, TMHMM, and TMPRED were used to identify possible membrane regions in the protein. A secondary structure was generated predicting 12 transmembrane spanning regions (TMS) (Figure 4). I-TASSER [25], [26] was used to develop a 3D model consisting of two domains of the first six, and last six TMS regions.

A sequence comparison including both respiratory and assimilatory NNPs identified conserved amino acids agreeing with previous analysis [3], [4], [27]. Site-directed mutagenesis was used to change 11 of the conserved amino acids to serine, threonine or glycine. Little is known about the bacterial nitrate transporters that play a role in nitrate assimilation rather than respiration. To provide more information on this group, ten amino acids conserved in assimilatory but not respiratory NNPs were also selected for mutagenesis. Amino acids were changed to either serine or to the amino acid present in the assimilatory transporter. Finally, NarK, the nitrate transporter of *E. coli* contains 5 cysteines and it was proposed these might play a role in inactivating nitrate transport in the presence of oxygen [28]. These cysteines are not conserved in the *M. tuberculosis* NarK2 which does however contain 2 cysteines which could fulfill a redox sensing role. These two were changed to serines. A total of 23 mutations were created (Table 2). Each mutant gene was constructed in the previously characterized *narK2* plasmid. The plasmids were then electroporated into the *M. tuberculosis narK2* knockout strain RVW3, where they integrated into the chromosome. The presence of the mutated copies of *narK2* were verified by Southern blot and DNA sequencing.

**Table 2:** Nitrate reductase activity of *narK2* mutants.

Strain	Aerobic^a^	NRP-1^b^	NRP-2^c^
WT	115±4	795±43	2365±61
RVW3	105±5	24±6	26±3
pNarK2	114±4	1012±36	2689±28
L8V	102±5	710±40	2192±16
F19S	102±10	326±26	1388±173
P50S	136±15	118±6	415±27
R58S	109±4	21±1	35±1
G69S	134±10	620±19	2083±29
G70P	118±6	792±49	2364±54
T78S	108±3	830±21	2318±38
P84T	111±4	678±21	1582±59
G89S	131±6	835±80	2621±167
A92G	108±3	830±47	2318±38
Y97S	122±9	993±84	2977±23
F114S	140±3	464±55	1340±102
I118L	126±11	975±100	2162±76
R129S	127±6	950±114	3179±53
G131S	104±3	56±8	254±7
G140S	125±12	355±35	1203±68
A145G	127±5	794±49	1951±280
Y215S	126±6	39±3	65±5
R259G	101±17	26±1	36±9
D267G	103±3	337±17	1258±36
C309S	111±4	797±35	2873±208
G315S	120±4	454±17	1364±47
C378S	108±2	800±39	2808±132

Mean nitrite concentration (micromolar) ± standard deviation.

a After 115 h of growth (final OD_580_ of ∼0.4).

b After 115 h in the Wayne model.

c After 255 h in the Wayne model.

All of the point mutation produced no effect on aerobic growth or the shiftdown growth curves in the Wayne model (Data not shown). Nitrite production was measured during aerobic growth to verify that nitrate reductase enzyme levels were similar in the mutants (Table 2). All mutants including the knockout RVW3 showed similar levels of nitrite production during aerobic culture.

Wild type *M. tuberculosis* and the complemented RVW3 pNarK2 strain produced low levels of nitrite during aerobic growth, with high levels during both microaerobic (NRP-1) and anaerobic (NRP-2) conditions (Table 2). However, many mutants as well as RVW3 show a defect during hypoxia. The strongest effect was seen with P50S, R58S, G131S, Y215S and R259S which were effectively inactive. Mutants F19S, G69S, P84T, F114S, F140S, D267G and G315S showed different levels of inhibition. Some mutations, including those based on assimilatory transporters, had no effect on nitrite levels. All mutants with reduced activity in NRP-1 showed a similar defect in NRP-2.

### Effect of each Mutation on Redox Control

**Figure 5:**
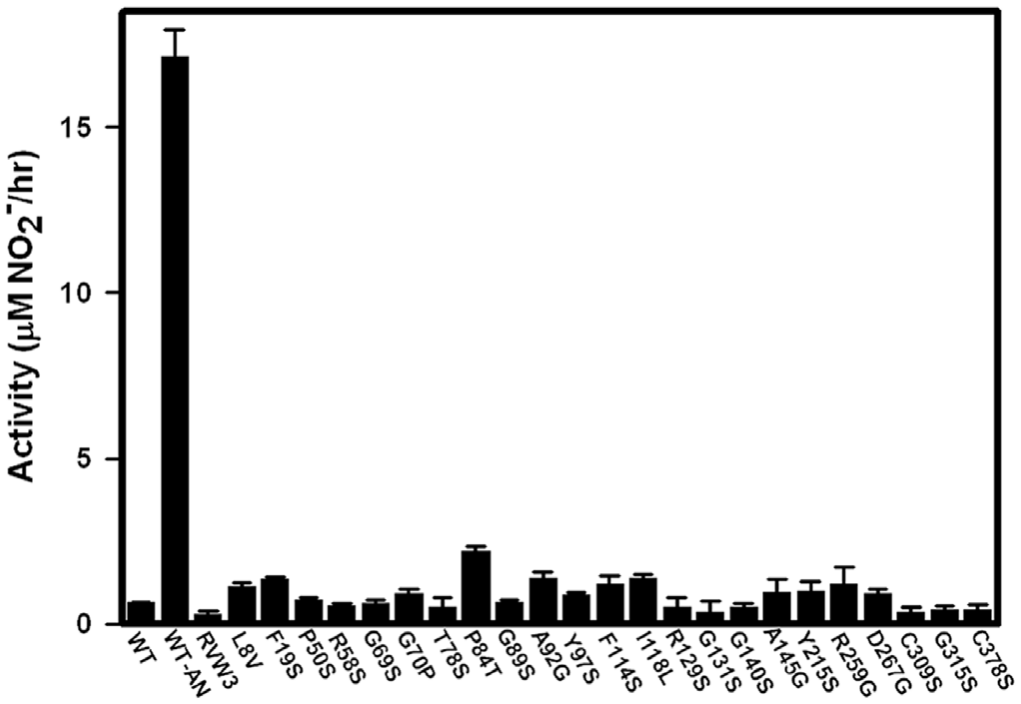
Oxygen inactivation of NarK2 mutants. The activity of each mutant was measured following exposure to oxygen. Wild type *M. tuberculosis* cultures incubated either anaerobically (WT-AN) or aerobically (WT) were included as controls. The standard deviation is indicated.

To determine if each transporter was still inactivated by oxygen a culture of each mutant was grown into NRP-1. It was opened, nitrate added and then nitrite measured at intervals over 5 hrs (Figure 5). The rate of nitrite production by wild type NarK2 was approximately 15-fold higher during anaerobiosis in comparison to aerobic conditions. Each mutant was inactive suggesting no role for these amino acids in the redox control of NarK2.

### Redox Control of Nitrate Transport

No mutants were identified that affected the inhibition of nitrite production by oxygen. Therefore the role of nitrate transport during oxygen inhibition was reexamined by analyzing the *M. tuberculosis* strain expressing the assimilatory *B. subtilis* NasA. Assimilatory nitrate transporters such as NasA function during aerobic growth and are not inactivated by the presence of oxygen. If this inactivation is due to a change in the proton gradient or other factor required for transport then NasA should also be inactivated when expressed in aerobic *M. tuberculosis*. If inactivation is due to NarK2 then NasA expressing cultures should show activity during aerobic incubation.

**Figure 6:**
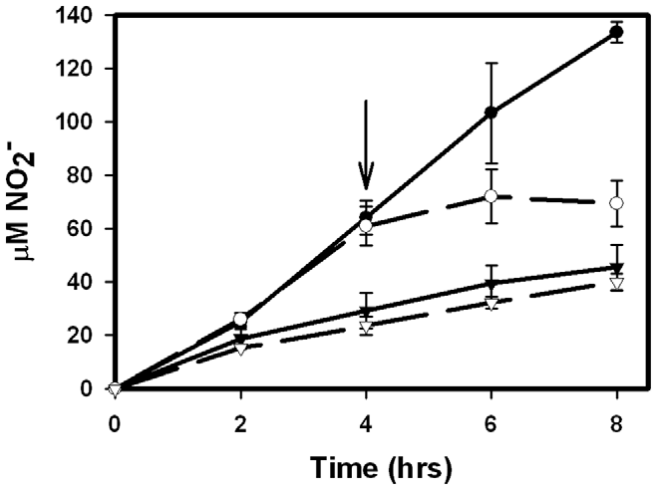
Oxygen inactivation of NarK2 but not NasA. Nitrite levels were determined in cultures of RVW3 pNarK2 (circles) and RVW3 pNasA (triangles). NRP-1 cultures were incubated anaerobically for 8 h (solid symbols), or anaerobically for 4 hrs, opened and then incubated aerobically for an additional 4 h (empty symbols). The arrow indicates when the cultures were shifted from anaerobic to aerobic conditions. The standard deviation is indicated.

Nitrite production was measured in a NasA expressing strain under aerobic conditions. RVW3 strains expressing either *narK2* or *nasA* were grown into NRP-1. Nitrate was added and each culture was then incubated anaerobically (Figure 6). As seen previously (Table 1) RVW3 expressing *narK2* produced higher levels of nitrite than RVW3 with *nasA* during anaerobic incubation although both genes were under the control of the *narK2* promoter. If an NRP-1 culture expressing NarK2 was exposed to air a rapid decrease in nitrite production occurred. This decrease was not seen in cultures expressing NasA. This suggests that the nitrate transporter NarK2 is the point of control for oxygen inhibition. The *nasA* strain also showed slightly higher nitrite production during aerobic growth due to low expression of *nasA* from a plasmid promoter (Table 1).

## Discussion

### Transport of Nitrate

*M. tuberculosis* has the ability to persist for decades in humans despite cell-mediated immunity. The initial encounter of the host with *M. tuberculosis* results in phagocytosis of the bacteria by macrophages in the alveoli. This is followed by replication of the bacteria within unactivated macrophages. Organized granulomas are formed as delayed type hypersensitivity develops, and the number of bacteria plateaus. In humans these tubercle lesions are able to control bacterial replication and isolate the bacteria but not eliminate them completely.

Two stresses that *M. tuberculosis* is exposed to in granulomas are hypoxia and nitric oxide. Nitric oxide produced by macrophages breaks down by a variety of pathways with nitrate being one stable end product. *M. tuberculosis* responds to both hypoxia and nitric oxide by initiating a complex developmental program resulting in a non-replicating persistent state characteristic of latent infection [8], [29]. The nitrate reductase system is induced to maintain the proton gradient for the continued production of ATP. This allows *M. tuberculosis* to survive the inhibition of respiration, and to provide energy during the development of the NRP state.

At physiological pH nitrate is an ion (pK_a_ of HNO_3_ =  −1.3) which, along with the membrane potential of *M. tuberculosis* at −110 mV [30], limits diffusion into the cell. Porins allow nitrate to cross the first permeability barrier of the outer lipid layer of *M. tuberculosis* [31]. NarK2 then transports nitrate across the cell membrane to the cytoplasm where the active site of nitrate reductase is located.

In *Paracoccus pantotrophus* two nitrate transport systems were identified [32]. One, identified as a Type I nitrate/proton symporter utilized the proton gradient and was active when nitrate was initially added to the medium. As intracellular nitrite levels increased the second transporter, a Type II nitrate/nitrite antiporter became active [4], [32]. The NarK2 protein of *M. tuberculosis* is a Type I NNP [3]. NarK2 of *M. tuberculosis* was sensitive to the protonophores DNP and FCCP (Figure 1), and no effect of nitrite on early nitrate reductase activity was detected (Figure 2). This suggests NarK2 is a H^+^/NO_3_^−^ symporter, and supports the proposal that the Type I subgroup are proton/nitrate symporters.

A nitrate/proton symporter requires one proton to import the nitrate substrate, which makes the process appear energetically unfavorable. The genome of *M. tuberculosis* encodes 3 additional homologues to *narK2* named *narK1*, *narK3* and *narU*. These 3 are predicted to be Type II nitrate/nitrite antiporters but none of them are induced during hypoxia [34], [35]. In addition, in the Wayne model the *narK2* knockout mutant showed only a low level of nitrate reductase activity attributed to diffusion suggesting NarK2 is the only transporter active under these conditions [15].

There are several factors that may explain why *M. tuberculosis* relies only on a H^+^/nitrate symporter. Although the reduction of nitrate in place of oxygen results in fewer protons exported [32] *M. tuberculosis* also responds to hypoxia *in vitro* and chronic infection in the mouse lung by switching from a proton-pumping to a non-proton-pumping NADH dehydrogenase [16]. Even in the presence of nitrate *M. tuberculosis* does not grow anaerobically, unlike *P. pantotrophus* and *E. coli* both of which use nitrate/nitrite antiporters during nitrate reduction. Without replication the energy demand of cells would greatly decrease. During anaerobiosis ATP synthesis and the proton motive force are still essential for survival [30], [33] but additional functions of nitrate reductase may be more important during NRP in *M. tuberculosis*. These include redox balancing by NADH/NAD^+^ recycling, and the use of the proton-utilizing nitrate reductase reaction to reduce internal acid stress.

**Table 3:** Comparison of the Effect of Mutations in NarK2 to Mutations in the Comparable Amino Acids in Other Nitrate/Nitrite Porters.

Strain	NarK2	NrtA^a^	NarU^b^	NarK^c^
L8V	No effect			
F19S	Inhibit	F47-Inhibit		
P50S	Inhibit			
R58S	Inactive	R87-Inhibit/Inactive	R66-Inactive	R66/R520-Inactive
G69S	Inhibit			
G70P	No effect		G99-No effect	
T78S	No effect		P113-No effect/Inhibit	
P84T	Inhibit			
G89S	No effect			
A92G	No effect			
Y97S	No effect			
F114S	Inhibit		F145-Inactive	
I118L	No effect			
R129S	No effect			
G131S	Inactive	G157-Inactive	G162-Inactive	
G140S	Inhibit	G167-Inhibit	G172-No effect/Inactive	
A145G	No effect	G172-Inhibit		
Y215S	Inactive		Y261-No effect	
R259G	Inactive	R368-Inhibit/Inactive		R269/R736-Inactive
D267G	Inhibit		D311-Inactive	
C309S	No effect			
G315S	Inhibit	G433-Inhibit		
C378S	No effect			

Mutations created in the *M. tuberculosis narK2* are compared to mutations in the comparable amino acid of other nitrate transporters. ‘Inactive’ indicates a protein with no activity, while ‘inhibit’ is defined as reduced activity. ‘No effect’ refers to wild type levels of activity. In examples where more than one mutation was made two results may be listed.

a Assimilatory NrtA of *Aspergillus nidulans* [27], [41].

b Respiratory NarU of *E. coli* [39].

c Respiratory NarK of *Paracoccus pantotrophus* [42].

Nitrite can be toxic especially in combination with the acidic conditions of the macrophage. Since it is not reduced further *M. tuberculosis* must export the nitrite which may require additional energy. A nitrate transporter is required for maximum nitrate reductase activity suggesting the export of the nitrite would also require a nitrite exporter. A nitrate/nitrite antiporter would seem ideal for this purpose but NarK2 does not fulfill this role. The identity of this nitrite exporter is not known.

### Mutagenesis of *narK2*

The 3D structures of several members of the major facilitator superfamily have been determined and are all very similar [36]–[38]. Extensive mutagenic studies of some members have allowed detailed structural and functional analysis of these transporters but no detailed structure has been determined for an NNP member. In this study a detailed mutagenic analysis of the NarK2 protein of *M. tuberculosis* was performed which included sites that had not previously been mutated. It is possible to compare these results to previous mutagenic studies involving NNPs (Table 3) [27], [39]–[43] and the 3D structure of related transporters.

A nitrate signature is present in all members of the NNP family including NarK2 [1], [27], [41], [44]. In NarK2 it is found in the predicted intracellular loop preceding and including membrane spanning region 5 (amino acids 125–144) (Figure 4). Based on the similarity to the GlpT and LacY transporters this section, along with TSM 2, 8 and 11, make up the inner substrate binding region [36], [38]. In NarK2 four residues in the signature were mutated, R129S, G131S, G140S and A145G. The mutation of glycine 131 produced an inactive protein, similar to the result seen with the nitrate transporter NarU of *E. coli* [39], and NrtA of *Aspergillus nidulans* [27](Table 3). The loss of G140 produced a partially active protein as was seen with this residue in NrtA [27]. The loss of the charged arginine at 129 had little effect suggesting the nitrate signature does not need this positive charge.

In the glycerol-3-phosphate transporter GlpT of *E. coli* two arginines bind the negatively charged phosphate of the substrate [38]. In NarK2 of *M. tuberculosis* two arginines (R58 and R259) were important for activity while a third, R129, was not. These two essential intramembrane arginines probably bind nitrate. Treatment of *Paracoccus denitrificans* with phenylglyoxal reduced nitrate transport suggesting an important role for arginine in NarK [45]. In both NarK of *Paracoccus pantotrophus* [42] and NrtA of *A. nidulans* [27], [41] the conserved arginines in TMS region 2 and 8 were identified as critical for activity. It was proposed the charged arginine directly interacts with nitrate [41]. Substitution of the arginines with positively charged lysines reduced, but did not eliminate, activity in NrtA.

Mutation F19S resulted in reduced activity, and the mutation of residue L8V had no effect on activity. These residues are in a region (amino acids 10–20) similar to a motif conserved in nitrate transporters of eukaryotes. This region has been proposed to regulate the pore opening by closing it after the substrate binds [27].

Major facilitator superfamily members also contain two conserved regions between membrane spanning regions 2 and 3, as well as between 8 and 9 [1]. This region is proposed to maintain the curvature of TMS2 and TMS8 [38]. The mutation of 2 glycines to serines in the first region (G69S and G70P) had little effect on nitrite production suggesting the importance of the glycines may be their small size. A similar change in NarU of *E. coli* also had little effect [39]. A change in the conserved aspartic acid in the second signature (D267G) resulted in decreased activity in NarK2 of *M. tuberculosis* as well as NarU of *E. coli* [39](Table 3).

NarK2 has a high glycine content (39 out of 395 amino acids). In particular the proposed TMS region 5 has five glycines and TMS 11 has six (Figure 4). Four of the most highly conserved glycine residues were changed to serines, and three of these mutants (G131S, G140S, G315S) had reduced activity. This could indicate a requirement for tight packing in the membrane. Not every conserved amino acid was required as the G69S mutation showed no apparent change in activity.

The conserved proline at position 84 was important for activity in both the *M. tuberculosis* NarK2 and the *E. coli* NarU, but the conserved tyrosine at 215 was important only in NarK2 [39]. Proline, like glycine, is often important for flexibility and this could explain the importance of the intramembrane prolines at position 84 and 50.

It is possible that some of the mutants created here resulted in reduced expression of the mutant NarK2. However in other mutational studies with nitrate transporters, no effect on expression was seen [27], [39]–[41]. Some of these mutations may affect insertion of NarK2 into the membrane although computer analysis did not identify any obvious changes in structure. However without an antibody to NarK2 it is not possible to determine protein levels or location at this time.

### Redox Effect

Nitrate reduction is regulated by control of nitrate transport in *M. tuberculosis*. Transcription of *narK2* is induced by hypoxia, while the activity of this transporter is controlled by redox levels in the cell [15], [17]. Even in the presence of oxygen, NarK2 remains active if respiration is inhibited, for example by nitric oxide [17]. Nitrate transporters may sense the redox state of the cell by interacting with the quinone pool [17], [21]. Quinone interacts with proteins at Q Sites which have only weak similarity making them difficult to predict [46], [47]. There are no obvious Q sites in the *M. tuberculosis* NarK2 (Data not show).

Transport of nitrate in *E. coli* was sensitive to N-ethylmaleimide which could indicate a role for redox sensitive sulfhydryl groups [48]. When the *E. coli narK* was sequenced the five cysteines that were encoded were proposed to play a role in redox control [28]. Mutating the two cysteines of NarK2 to serines resulted in no change in activity indicating they are not essential for function. In the assimilatory nitrate transporter *of Aspergillus nidulans*, NrtA none of the eight cysteines were essential [40]. In NarK2 both cysteine mutants were inactive in the presence of oxygen suggesting no role in redox inactivation (Figure 5).

None of the mutants showed significant activity in the presence of oxygen. NasA however was active showing that NarK2 is the point of redox inactivation (Figure 6). The reason for the dual hypoxic control of *narK2*, transcription and activity, is unknown. There was no noticeable defect in the strain expressing the oxygen insensitive NasA. Growth, shiftdown and recovery from hypoxia were all similar to the *narK2* expressing strain (Data not shown).

## Supporting Information

Table S1 **Primers used in this study**
(DOC)
