# Supplementary material for: Mutational Analysis of the Respiratory Nitrate Transporter NarK2 of Mycobacterium tuberculosis
Source: PLoS One. 2012 Sep 18;7(9):e45459. doi: 10.1371/journal.pone.0045459 (PMC3445494; doi:10.1371/journal.pone.0045459)
Supplement: Table S1 — Primers used in this study (DOC) [file pone.0045459.s001.doc]

**Table S1. Primers used**

| **Name** | **Sequence (5’🡪3’)** |
| --- | --- |
| pNasA-f | AAAATAATGAGGGTACCGAAAGGGGAATCT |
| pNasA-r | TATCTAGATCGGTTCACTGTTGAAAATCAA |
| p173 | AGATTATCAAAAAGGATCTTCACCTAGATC |
| p174 | ACAGCGGTAAGATCCTTGAGAGTTTTCGCC |
| L8V-f | GAGAGGGCAAGCGGCCAATGTCGTGCTGGCCACCTGGATCTCG |
| L8V-r | CGAGATCCAGGTGGCCAGCACGACATTGGCCGCTTGCCCTCTC |
| F19S-f | GGATCTCGGTGGTCAACTCCTGGGCGTGGAACCTGATC |
| F19S-r | GATCAGGTTCCACGCCCAGGAGTTGACCACCGAGATCC |
| P50S-f | GCGTCGCTGCTCGTCGCCACCTCGATCCTGGTGGGTGCCCTTGG |
| P50S-r | CCAAGGGCACCCACCAGGATCGAGGTGGCGACGAGCAGCGACGC |
| R58S-f | CTGGRGGGTGCCCTTGGCACGATCGTCACCGGGCCGCTC |
| R58S-r | GAGCGGCCCGGTGACGATCGTGCCAAGGGCACCCACCAG |
| G69S-f | CCGCTCACCGACCGCTTCTTCGGGCGCGCCATGCTCATC |
| G69S-r | GATGAGCATGGCGCGCCCGAAGAAGCGGTCGGTGAGCGG |
| G70P-f | CCGCTCACCGACCGCTTCGGCCCGCGCGCCATGCTCATCGCGGTG |
| G70P-r | CACCGCGATGAGCATGGCGCGCGGGCCGAAGCGGTCGGTGAGCGG |
| T78S-f | GCGCCATGCTCATCGCGGTGTCGCTGGCGTCGATCCTCCCGGTG |
| T78S-r | CACCGGGAGGATCGACGCCAGCGACACCGCGATGAGCATGGCGC |
| P84S-f | ACGCTGGCGTCGATCCTCACGGTGCTCGCGGTGGGG |
| P84S-r | CCCGACCGCGAGCACCGTGAGGATCGACGCCAGCGT |
| G89S-f | ATCCTCCCGGTGCTCGCGGTCTCGGTCGCGGCAACCATGGGCTCCTACG |
| G89S-r | CGTAGGAGCCCATGGTTGCCGCGACCGAGACCGCGAGCACCGGGAGGAT |
| A92G-f | GTGCTCGCGGTCGGGGTCGCGGGAACCATGGGCTCCTACGCGTTG |
| A92G-r | CAACGCGTAGGAGCCCATGGTTCCCGCGACCCCGACCGCGAGCAC |
| Y97S-f | GTCGCGGCAACCATGGGCTCCTCCGCGTTGCTGGTGTTTTTCGGG |
| Y97S-r | CCCGAAAAACACCAGCAACGCGGAGGAGCCCATGGTTGCCGCGAC |
| F114S-f | GTTGCCGGCACGATCTCCGCCGTCGGCATCCCGTTC |
| F114S-r | GAACGGGATGCCGACGGCGGAGATCGTGCCGGCAAC |
| I118L-f | GGCACGATCTTCGCCGTCGGCCTCCCGTTCGCCAACAACTGGTACCAG |
| I118L-r | CTGGTACCAGTTGTTGGCGAACGGGAGGCCGACGGCGAAGATCGTGGC |
| R129S-f | AACTGGTACCAGCCGGCGTCGCGCGGTTTCTCCACCGGCGTGTTCGG |
| R129S-r | CCGAACACGCCGGTGGAGAAACCGCGCGACGCCGGCTGGTACCAGTT |
| G131S-f | TACCAGCCGGCGCGGCGCTCTTTCTCCACCGGCGTC |
| G131S-r | CACGCCGGTGGAGAAAGAGCGCCGCGGCTGGTA |
| G140S-f | GGCGTGTTCGGTATGTCCATGGTCGGCACCGCG |
| G140S-r | CGCGGTGCCGACCATGGACATACCGAACACGCC |
| A145G-f | GGTATGGGCATGGTCGGCACCGGGCTCTCGGCGTTCTTCACCCCG |
| A145G-r | CGGGGTGAAGAACGCCGAGAGCCCGGTGCCGACCATGCCCATACC |
| Y215S-f | TGGGAGATGTCGTTTCTGTCCGCGATCGTGTTCGGCGGG |
| Y215S-r | CCCGCCGAACACGATCGCGGACAGAAACGACATCTCCCA |
| R259S-f | GCGGCGGTGCTGGCCGGGCCGGTGGGCGGGTGG |
| R259S-r | CCACCCGCCCACCGGCCCGGCCAGCACCGCCGC |
| D267S-f | GTGGGCGGGTGGCTCTCCGGCCGGATCGCACCGAGGCAC |
| D267S-r | GTCCCTCGGTGCGATCCGGCCGGAGAGCCACCCGCCCAC |
| G309S-f | CATCACCCTGGCGGTCTGTCTCGGCGTGGGCACCGG |
| G309S-r | CCGGTGCCCACGCCGAGACAGACCGCCAGGGTGATG |
| G315S-f | CTCGGCGTGGGCACCTCCGGCGTGTTCGCGTGG |
| G315S-r | CCACGCGAACACGCCGGAGGTGCCCACGCCGAG |
| C378S-f | GACCGCGGTCGCGTCTACCTACACCGCGCTGCACGCG |
| C378S-r | CGCGTGCAGCGCGGTGTAGGTAGACGCGACCGCGGTC |

Mismatches used to create mutations are indicated by underlining
